# Supplementary figures and images for: Dynamic longitudinal behavior in animals exposed to chronic social defeat stress
Source: PLoS One. 2020 Jul 23;15(7):e0235268. doi: 10.1371/journal.pone.0235268 (PMC7377442; doi:10.1371/journal.pone.0235268)

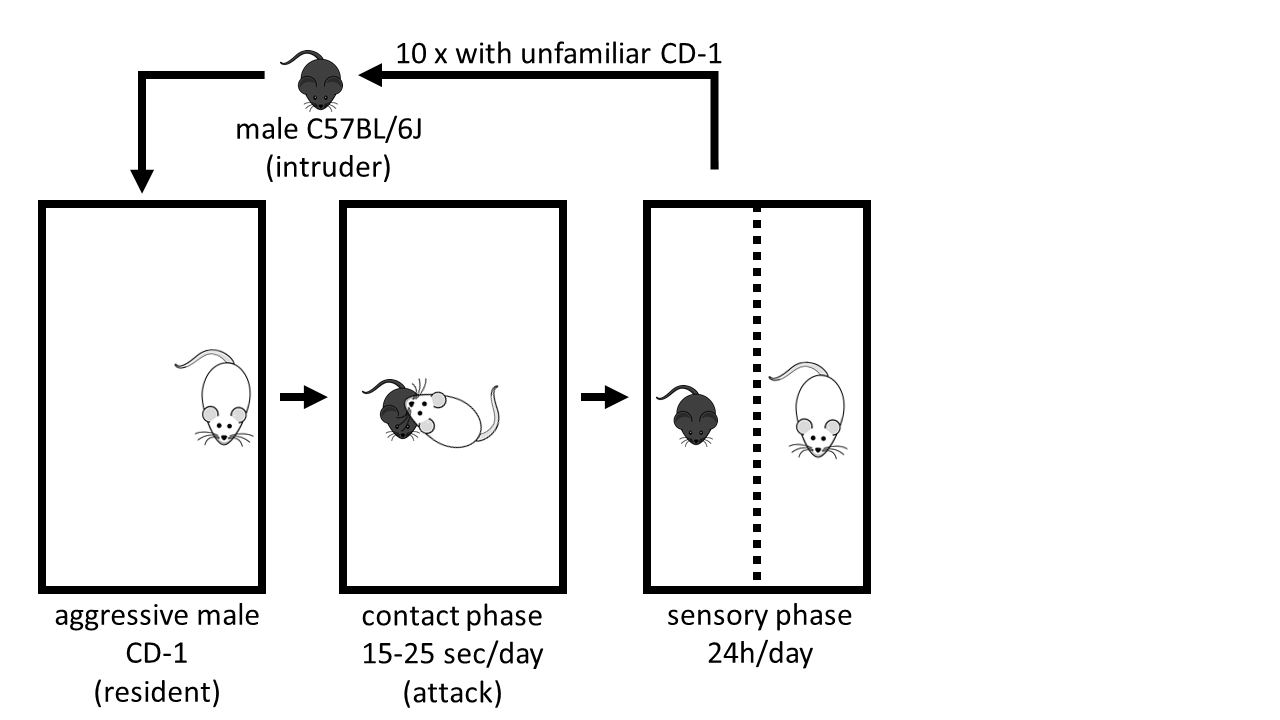

Supplement: S1 Fig — (TIF) [file pone.0235268.s002.tif]

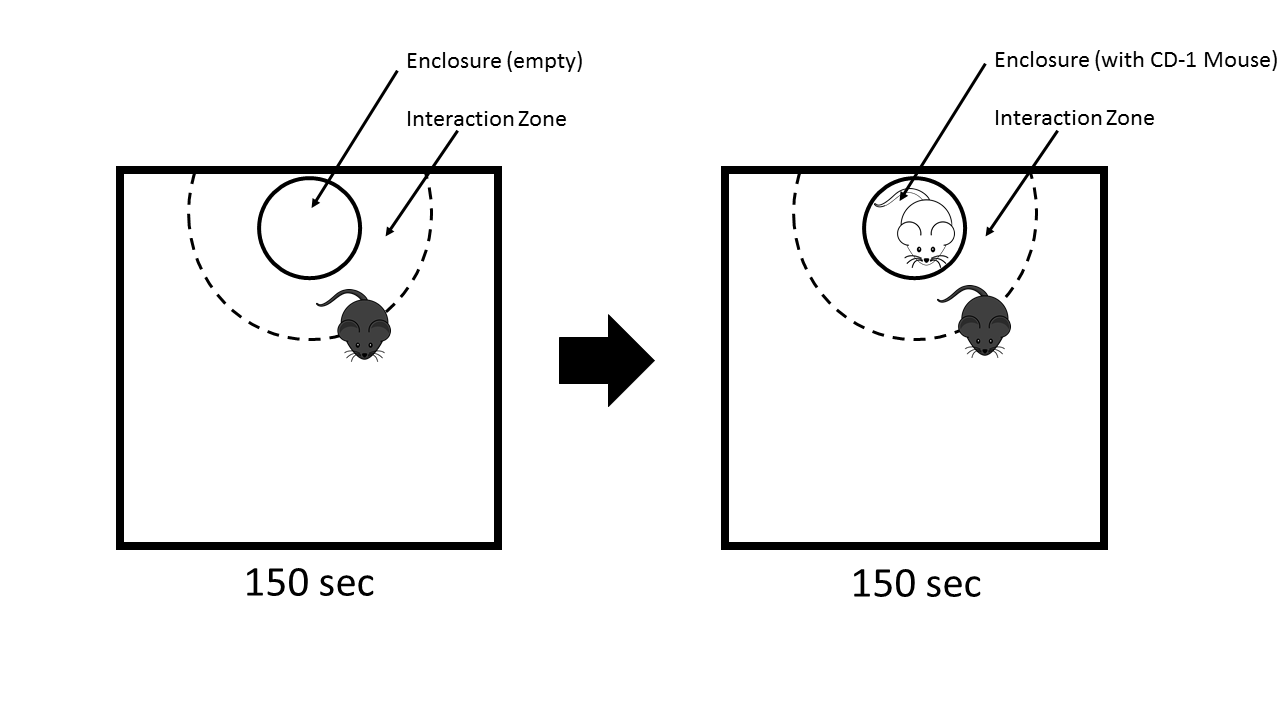

Supplement: S2 Fig — (TIF) [file pone.0235268.s003.tif]

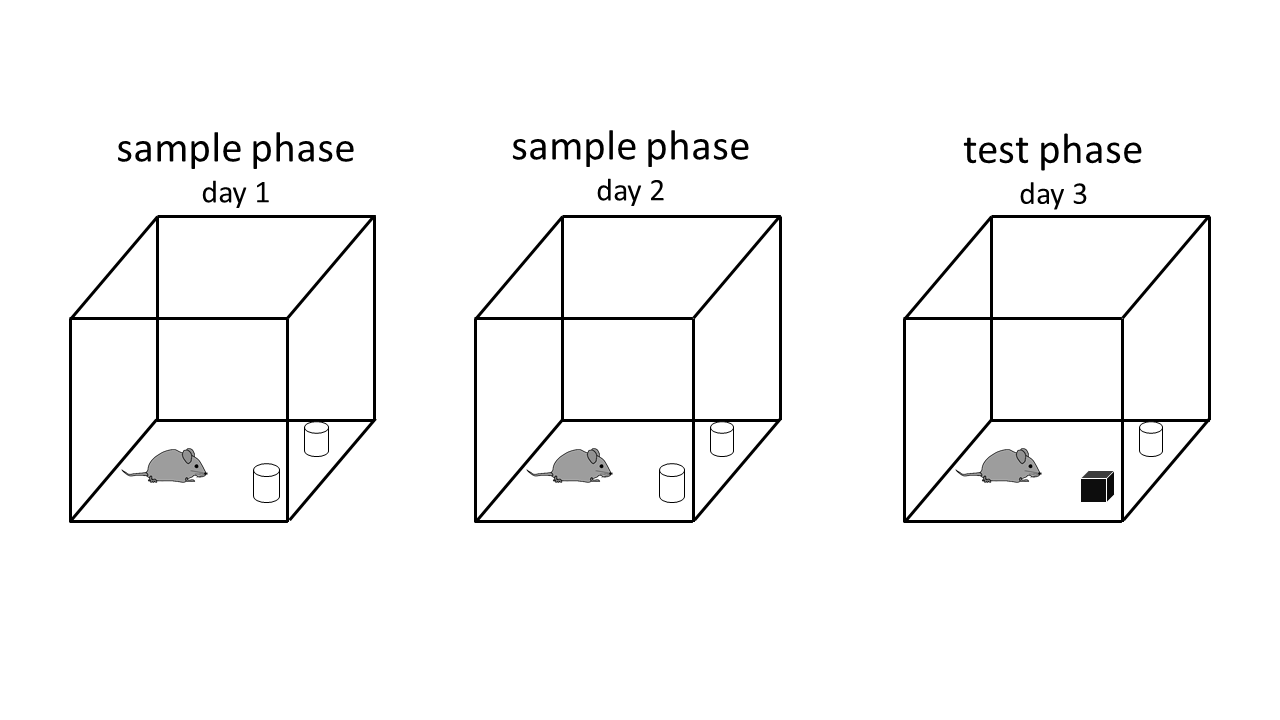

Supplement: S3 Fig — (TIF) [file pone.0235268.s004.tif]

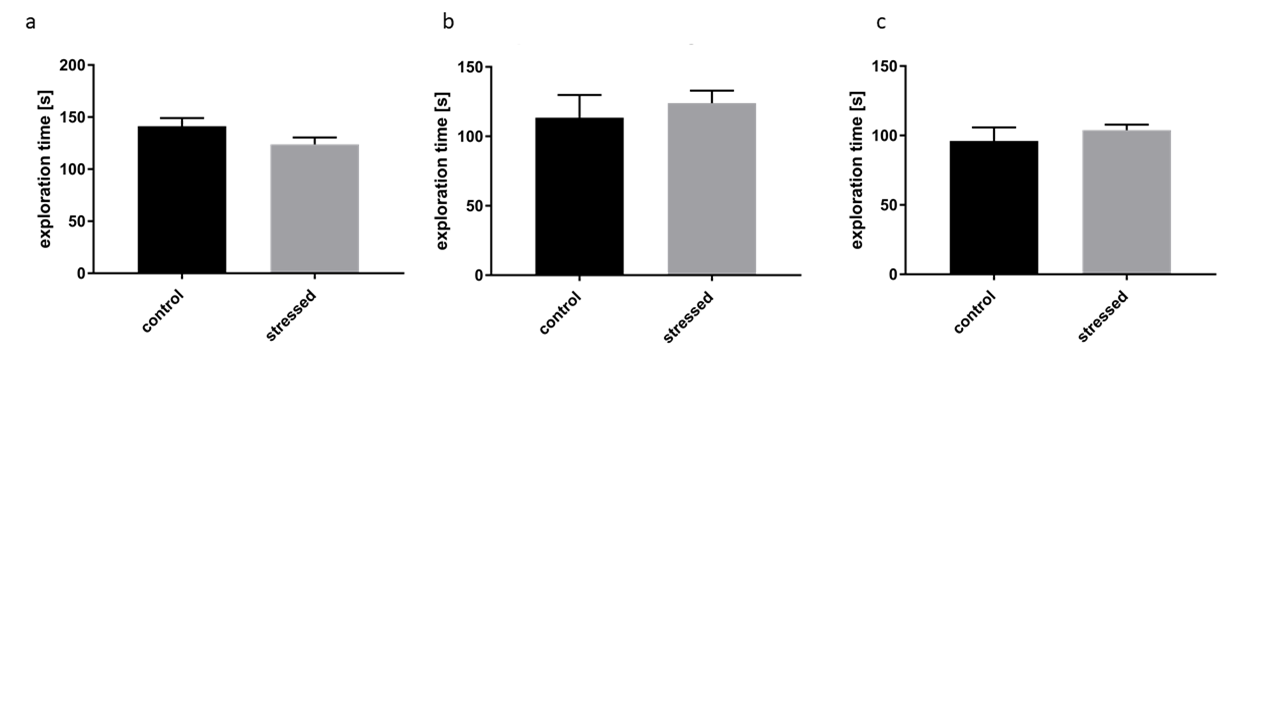

Supplement: S4 Fig — (a) 24 hours post exposure. (p = 0.1641, unpaired t-test with Welch’s correction) (b) 8–10 days post exposure. (p = 0.5838, unpaired t-test with Welch’s correction) (e) 22–24 days post exposure (p = 0.7836, unpaired t-test with Welch’s correction). Data are shown as mean + standard error of the mean. (TIF) [file pone.0235268.s005.tif]

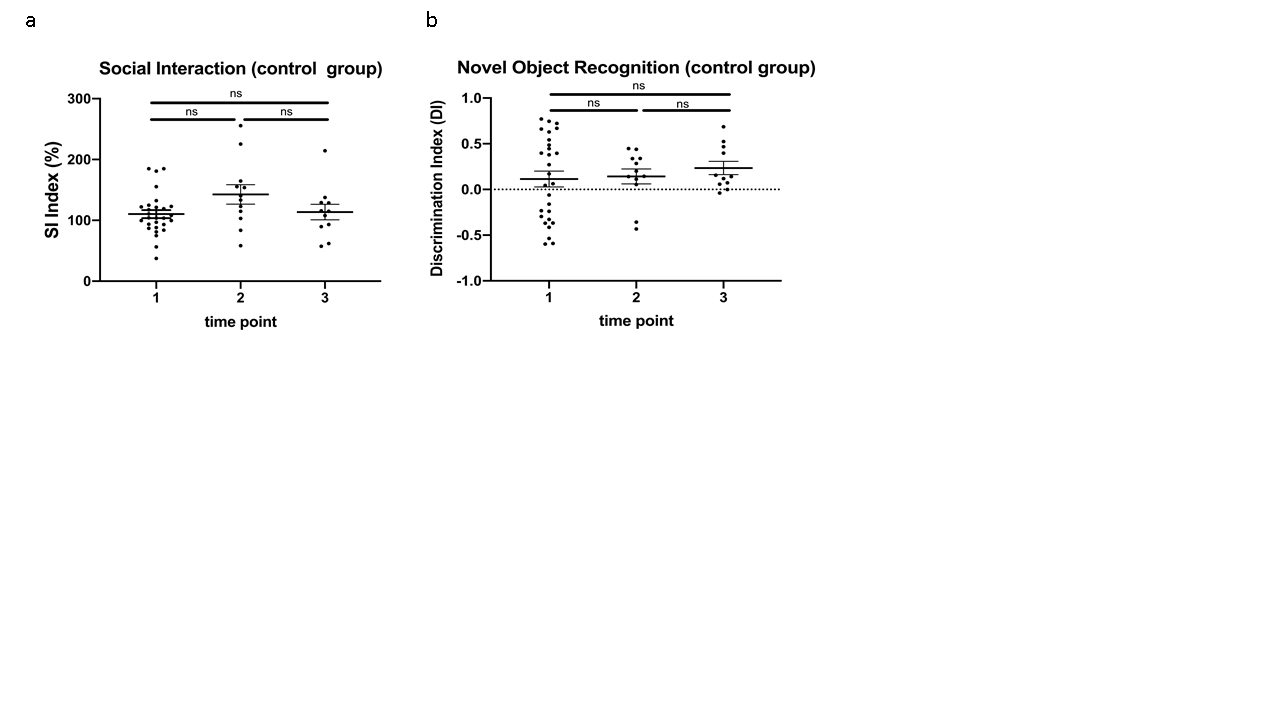

Supplement: S5 Fig — (a) SI scores of control mice in direct comparison revealed no significant differences (T1 vs. T2 p = 0.0898, T1 vs. T3 p = >0.9999, T2 vs. T3 p = 0.3114, 1-way ANOVA with Bonferroni post hoc test) (b) DI scores of control mice in direct comparison shows no significant difference in cognitive skills at three timepoints (T1 vs. T2 p = >0.9999, T1 vs. T3 p = >0.9999, T2 vs. T3 p = >0.9999, Kruskal-Wallis test and Dunn’s post hoc test). (TIF) [file pone.0235268.s006.tif]

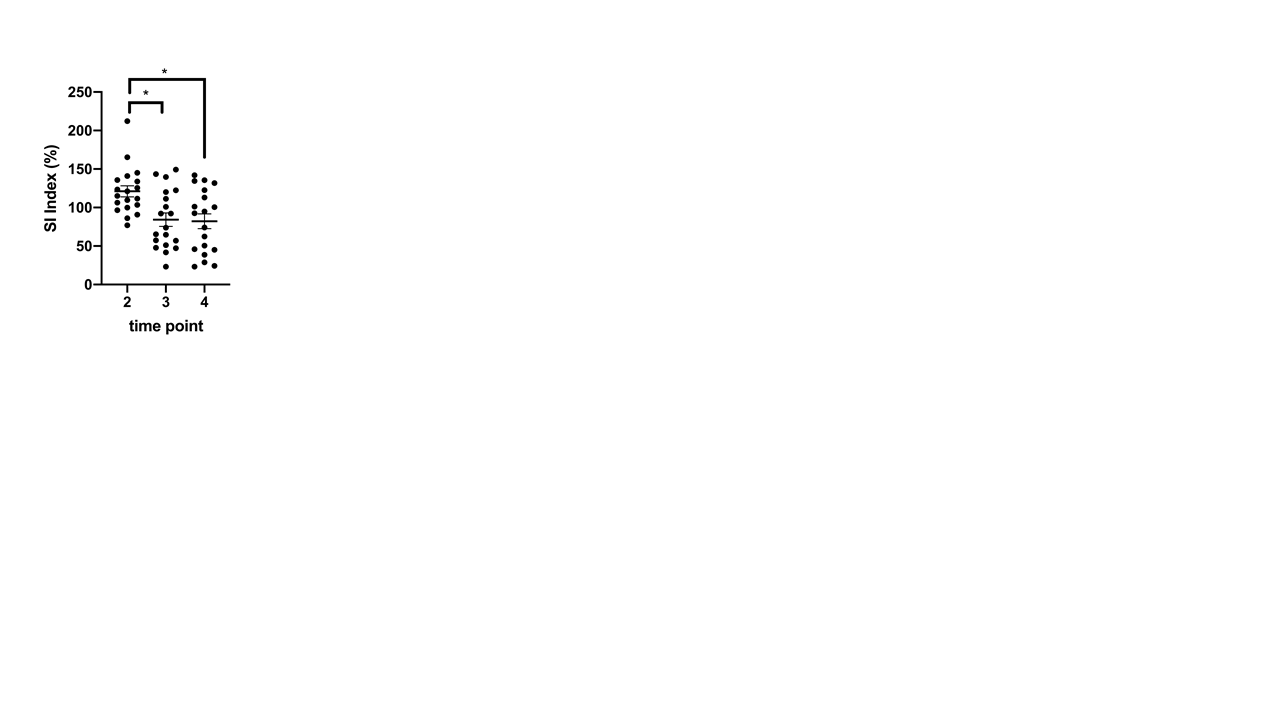

Supplement: S6 Fig — SI scores were significantly lower at T3 and T4 compared to T2 (T2 vs. T3 p = 0.0296, T2 vs. T4 p = 0.0210, T3 vs. T4 p = >0.9999, Kruskal-Wallis test and and Dunn’s post hoc test). (TIF) [file pone.0235268.s007.tif]

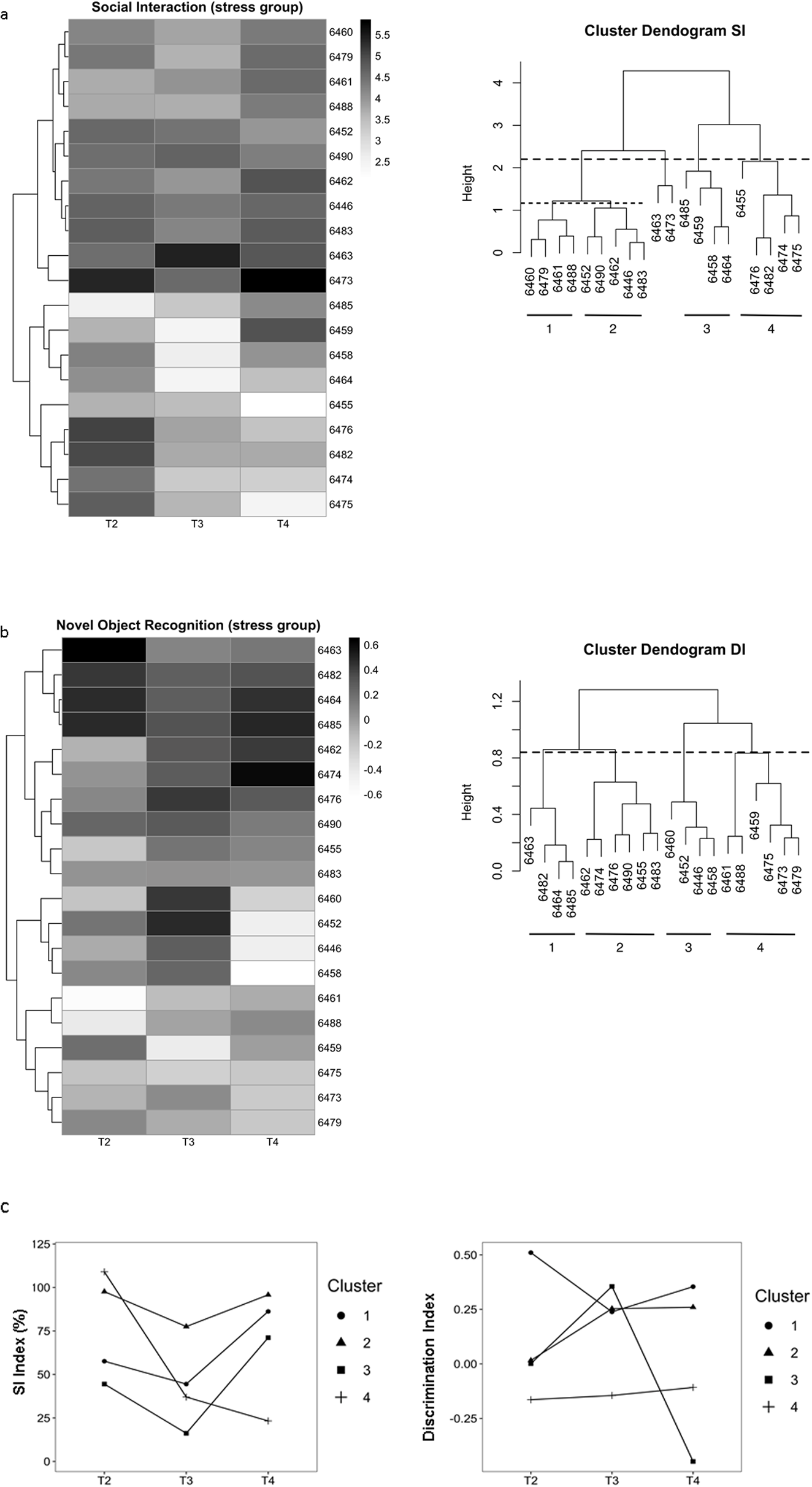

Supplement: S7 Fig — (a) Heatmap with hierarchical clustering (left panel) and corresponding dendogram (right panel) for SI scores of individual stressed animals at T2-T4. SI scores are shown on a log-scale. (b) Heatmap with hierarchical clustering (left panel) and corresponding dendogram (right panel) for DI scores of individual stressed animals at T2-T4. DI scores are shown on a log-scale. Repeated measures one-way ANOVA revealed that time significantly impacted the average SI scores of the stress group (p = 0.025). Bonferroni’s post hoc test confirmed that the average SI score was significantly reduced at T3 compared to T2 (p = 0.004). In contrast, average DI scores of the stressed group did not change over time (repeated measures one-way ANOVA, p = 0.152). (c) Average SI and DI scores at each time point for the 4 clusters of animals obtained using hierarchical clustering. (TIF) [file pone.0235268.s008.tif]

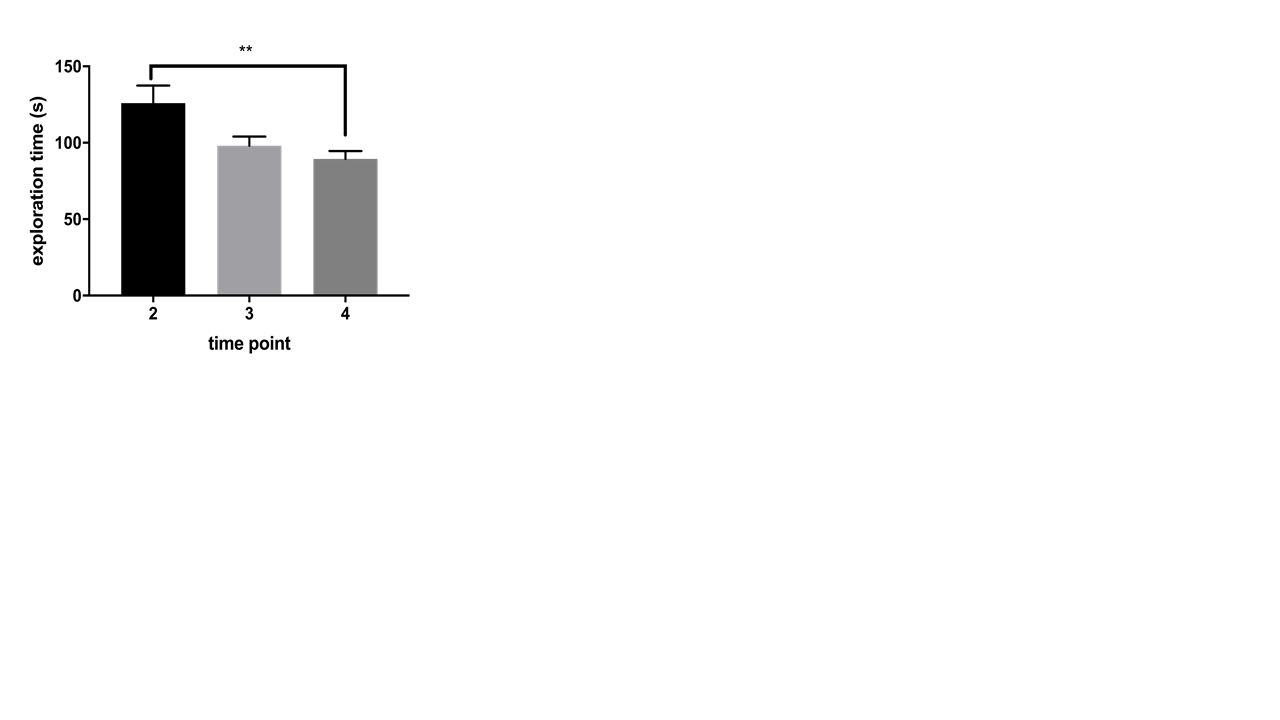

Supplement: S8 Fig — T2: 8–10 days post exposure. T3: 22–24 days post exposure. T4: 43–45 days post exposure (T2 vs. T3 p = 0.0551, T2 vs. T4 p = 0.0071, T3 vs. T4 p>0.9999, ANOVA and Bonferroni’s multiple comparisons test). Data are shown as mean + standard error of the mean. (TIF) [file pone.0235268.s009.tif]
